# Genetic Engineering for Global Food Security: Photosynthesis and Biofortification

**DOI:** 10.3390/plants8120586

**Published:** 2019-12-09

**Authors:** Andrew John Simkin

**Affiliations:** Genetics, Genomics and Breeding, NIAB EMR, East Malling, Kent ME19 6BJ, UK; andrew.simkin@emr.ac.uk; Tel.: +44-01732-52-3748

**Keywords:** photosynthesis, biofortification, yield, nutrition

## Abstract

Increasing demands for food and resources are challenging existing markets, driving a need to continually investigate and establish crop varieties with improved yields and health benefits. By the later part of the century, current estimates indicate that a >50% increase in the yield of most of the important food crops including wheat, rice and barley will be needed to maintain food supplies and improve nutritional quality to tackle what has become known as ‘hidden hunger’. Improving the nutritional quality of crops has become a target for providing the micronutrients required in remote communities where dietary variation is often limited. A number of methods to achieve this have been investigated over recent years, from improving photosynthesis through genetic engineering, to breeding new higher yielding varieties. Recent research has shown that growing plants under elevated [CO_2_] can lead to an increase in Vitamin C due to changes in gene expression, demonstrating one potential route for plant biofortification. In this review, we discuss the current research being undertaken to improve photosynthesis and biofortify key crops to secure future food supplies and the potential links between improved photosynthesis and nutritional quality.

## 1. Introduction

Over the last 70 years, agricultural yields have risen at a level that meets global demand. Most of these increases came about from the breeding of higher yielding varieties and advances in agronomic approaches [1], as well as improving yields by exploiting the natural photosynthetic variation in key crops [2,3]; however, more recently the yields of several important food crops have plateaued. By 2050, it is expected that the global population will exceed 9 billion [4,5]. To meet the ever-growing requirements for both food and fuel, it will be necessary to develop new varieties of crops with higher yields [6,7,8,9]. It has been estimated that a 70% to a 100% increase in the yield of crops such as wheat, soybean (*Glycine max*) and maize (*Zea mays*) will be required to meet these needs [10,11,12]. One avenue, clearing land for crop production, has the significant drawback of causing environmental damage to unique habitats, resulting in a global loss in biodiversity [13,14,15,16]. Increasing cultivatable land also comes at the cost of increased fertiliser and pesticide use which creates further damage to both marine and freshwater ecosystems [17] and results in a significant increase in greenhouse gas emissions [18]. To mitigate environmental damage, it will be necessary to meet global food demands without increasing the amount of cultivatable land. To increase yields whilst protecting the environment, the development of new varieties of key crops is essential. This may be achieved through improving photosynthesis through genetic manipulation (Section 2).

In addition to increasing crop yields, there is also a need to improve the nutritional quality. Over the last 5 decades, new crop varieties have increased production of calorie dense staple crops but the production of non-staple micronutrient-rich crops has not increased. Additionally, the food prices of micronutrient-rich non-staple crops such as fruits and vegetable and important animal products have increased, leaving poorer communities financially unable to obtain the variability needed for a healthy diet [19]. Globally, micronutrient deficiencies affect more than two billion people [4]. Micronutrient deficiencies are the direct result of poor diet where vitamins and minerals are below the threshold to sustain a healthy lifestyle [19]. Through biofortification (Section 3), there is potential to improve the bioavailability of important nutrients in new crops.

In this review, I will discuss a multi-targeted approach to increasing photosynthetic efficiency and a targeted approach to the generation of multi-vitamin crops and the potential links between boasting photosynthesis and impact on the final vitamin content of modified crops.

## 2. Photosynthesis

**Figure 1:**
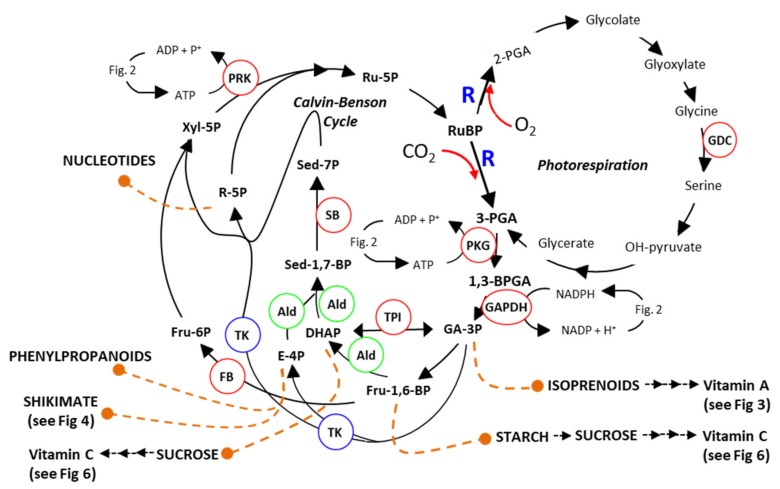
Schematic Representation of Photosynthesis and Photorespiration. Sedoheptulose-1,7-bisphosphatase (SB: EC.3.1.3.37), fructose-1,6-bisphosphate aldolase (Ald: EC.4.1.2.13), fructose-1,6-bisphosphatases (FB: EC.3.1.3.11), triosephosphate isomerase [TPI: EC 5.3.1.1], transketolase (TK: EC.2.2.1.1), phosphoribulokinase (PRK: EC.2.7.1.19), phosphoglycerate kinase (PGK: EC.2.7.2.3), ribulose-bisphosphate carboxylase (R: EC.4.1.1.39), Glyceraldehyde-3-phosphate dehydrogenase (GAPDH) and Glycine decarboxylase (GDC). (see [39]).

Photosynthesis is responsible, either directly through plant growth or indirectly through the food chain, for all the food consumed worldwide. Furthermore, all the fuel derived from oil was originally derived from photosynthates and accounts for as much as 90% of current energy production [20]. Crop yield is determined by the collective rate of photosynthesis over the growing season. A key determinant of this is the efficiency at which light is A, captured and B, converted into yield (i.e., biomass or grain). Carbon assimilation in plants involves two closely associated pathways, 1, the Calvin–Benson cycle (CBC) and 2, Photorespiration. These pathways are centred on the catalytic activity of Rubisco (ribulose-1,5-bisphosphate carboxylase/oxygenase; R; Figure 1), a complex stromal enzyme which accounts for more than 50% of all leaf protein and 25% of leaf nitrogen. Found in all photosynthetic organisms, Rubisco has been identified as a target for genetic manipulation and has thus been widely reviewed to date [21]. Enhancing photosynthesis, electron transport and photorespiration has been accepted as a key target for increasing crop productivity [22,23,24]. A recent comprehensive review by Simkin et al. [23] has touched upon this potential. In this review, we will focus on the impact of a multi-target approach to improving photosynthesis and potential impacts on nutrient value of transformed crops. 

### 2.1. Manipulation of Calvin–Benson Cycle

The CBC is subdivided into three stages, carboxylation, reduction and regeneration. The cycle, elucidated by Melvin Calvin et al. in the 1950s [25,26], involves 11 enzymes, which catalyse 13 different steps (Figure 1). Transgenic studies have demonstrated any decrease in any one of the key enzymes of the CBC has a significant negative impact on the rate of carbon assimilation and plant growth. These studies have shown that reductions in sedoheptulose-1,7-bisphosphatase (SBPase) [27,28,29], fructose-1,6-bisphosphate aldolase (FBPA) [30,31], fructose-1,6-bisphosphatases (FBPase) [32,33,34] or phosphoribulokinase [35] resulted in a decrease in final biomass yield. The results of these experiments showed that there are multiple limiting steps within the CBC and that increasing enzyme activities at any of these branch points has the potential to boost photosynthetic efficiency and as a consequence increase yields [36,37]. Furthermore, [CO_2_] enrichment experiments in field-based studies demonstrated that increasing CO_2_ assimilation results in increases in plant biomass [38]. 

Early work targeting single steps in the CBC was used to test the potential for increasing the rates of CO_2_ assimilation and evaluating their effects on plant development and biomass yield. For example, the over-expression of SBPase in tobacco (*Nicotiana tabacum*) [40,41], Arabidopsis (*Arabidopsis thaliana*) [42] and tomato (*Solanum lycopersicum*) [43] has shown that an increase in SBPase enzyme activity results in significant increases plant biomass (>30% increase in biomass). In follow-up work, increased SBPase activity in tobacco grown in open-air elevation of [CO_2_] experiments led to substantial increases in biomass yield [44]. More recently, the over-expression of SBPase in wheat, an important food crop, resulted in an increase in biomass yield and more importantly, a 30% to 40% increase in seeds yield [45]. Similar results were obtained by the over-expression of another CBC enzyme, FBPA, where an increase in biomass of 70–120% was observed under elevated [CO_2_] (700 ppm) [46]. However, recent work in tobacco showed that the over-expression of some CBC enzymes is not beneficial. The over-expression of transketolase, for example, led to a negative effect on plant development and plants regularly showed a chlorotic phenotype and retarded growth [47].

Work showing that the manipulation of individual enzymes can result in an increase in yield has been further investigated through the targeting of multiple enzymatic steps in CBC. For example, the over-expression of SBPase and FBPA in transgenic tobacco resulted in a cumulative increase in biomass yield (+62%) compared to plants expressing SBPase alone (+34%) [41], demonstrating that multigene manipulation of the CBC can lead to additional increases in yield. However, over-expression of SBPase and FBPA in transgenic Arabidopsis did not lead to a synergistic effect and the observed increases in biomass were similar between SBPase, FBPA and SBPase + FBPA expressing lines [42]. These contradictory results indicate that different targeted manipulations will be needed, depending on the plant species under evaluation. Moreover, photosynthetic carbon assimilation is a complex mechanism involving not just the CBC but photorespiration and photosynthetic electron transport. 

### 2.2. Can Increasing Photorespiration Increase Yield?

The carboxylation reactions carried out by R as part of the CB cycle, incorporating CO_2_ into complex downstream products, is contending with a parallel competing reaction where R fixes O_2_ in place of CO_2_ (Figure 1). This reaction, known as photorespiration, accounts for approximately 25% of the reactions carried out by R and leads to the formation of a molecule of 3-phosphoglycerate (3PGA) and a molecule of 2-phosphoglycolate (2PG). The formation and recycling of 2PG comes at a cost, impacting on photosynthetic efficiency and therefore negatively impacting yield potential [1,48,49,50,51,52,53,54,55]. A number of previous studies have shown that a reduction in photorespiratory flux under high photorespiratory conditions (i.e., high temperature or water stress) results in an overall reduction in photosynthetic efficiency and CO_2_ assimilation [56,57,58,59]. In contrast, green tissue-specific over-expression of the glycine decarboxylase (GDC) H-protein or L-protein has been shown to increase vegetative biomass in Arabidopsis and tobacco [42,60,61,62,63]. It is believed that increasing photorespiration prevents the build-up of toxic photorespiratory intermediates (e.g., 2-PGA, glycolate, glyoxylate or glycine) [42,53,60,62,64,65,66,67,68,69]. For example, 2-PGA has been shown to inhibit triose-phosphate isomerase (TPI) in pea [53], phosphoribulokinase (PRK) in spinach [64] and SBPase in Arabidopsis [70]. Furthermore, Glyoxylate inhibits ribulose-bisphosphate carboxylase/oxygenase (R) activation [68,69,71]. The over-expression of GDC-H was also recently shown to increase photosynthetic electron transport rates in Arabidopsis [42]. 

### 2.3. Enhancing Photosynthetic Electron Transport Increases Yield

**Figure 2:**
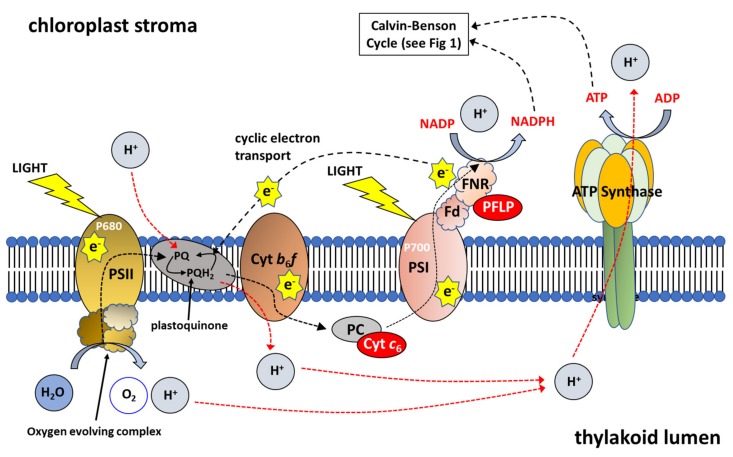
Schematic Representation of Photosynthetic Electron Transport. Photosystem I (PSI), Photosystem II (PSII), Cytochrome b6f complex (Cyt *b*_6_*f*), plastocyanin (PC), Ferredoxin (Fd) and ferredoxin-NADP reductase (FNR). Cytochrome *c*_6_ (Cyt *c*_6_) transfers electrons from the Cyt *b*_6_*f* complex to PSI at a faster rate than observed for PC; plant ferrodoxin-like protein (PFLP) (Adapted from [23]).

Photosynthetic electron transport chain (ETC) is a succession of complexes that moves electrons between photosystem I (PSI) and photosystem II (PSII), creating an electrochemical proton gradient needed for the synthesis of ATP (see [72] and references therein). Directly increasing photosynthetic electron transport by the expression of the algal (*Porphyra yezoensis*) cytochrome *c*_6_ has also been shown to increase yields in Arabidopsis [73,74] (Figure 2). Cytochrome *c*_6_ has been shown to replace plastocyanin (PC) as an electron carrier in cyanobacteria and green algae in response to copper deficiency [75]. Furthermore, in Arabidopsis, cyt *c*_6_ has been shown to transfer electrons from the cytochrome (cyt) *b*_6_*f* complex to PSI in vivo and faster than the reported rates for the native PC [73].

Furthermore, over-expression of the RieskeFeS protein in Arabidopsis, a key protein in the cyt *b*_6_*f* complex, leads to a substantial increase in the rates of CO_2_ assimilation, contributing to up to a 72% increase in biomass and a 51% increase in seed yield in the best line [76]. Using Chlorophyll fluorescent imaging and Dual-PAM measurements, these authors showed that the over-expression of RieskeFeS (and resulting increases in other proteins of the cyt *b*_6_*f* complex: cyt *b*_6_, cyt *f*, LhcaI, PsaA, PsbA, PsbD; see Figure 2), resulted in an increase in the potential quantum yield of PSI and PSII [76]. 

More recently, the over-expression of RieskeFeS in the C4 species *Setaria viridis* resulted in better light conversion in PSI and PSII with a 10% increase in complex content corresponding to a 10% increase in carbon assimilation [77]. Taken together, these data have suggested that the electron transport chain is also a limiting step in photosynthesis. Support for this came from antisense studies that have previously demonstrated that reductions in RieskeFeS protein levels result in decreases in photosynthetic electron transport and reduction in biomass and seed yield [78,79,80,81,82,83,84]. 

Other studies have looked at the over-expression of ferrodoxin (Fd), one of the final acceptors of electrons in the transport chain (Figure 2). Increasing the capacity for ferrodoxin to receive electrons would increase the capacity for photochemical quenching. However, constitutive OE of ferrodoxin led to no increase in photosynthetic efficiency in tobacco and no change to biomass yield was observed [85]. Furthermore, constitutive over-expression of ferrodoxin NADP reductase (FNR) in tobacco, the enzyme enabling the transfer of electrons from ferrodoxin to NADP+ (Figure 2) also led to no increase in photosynthetic efficiency or biomass yield [86]. More recently, constitutive OE of a plant ferrodoxin-like protein (PFLP), cloned from sweet peppers, in rice resulted in significantly higher electron transport rates, and up to a 1.3-fold increase in the rates of photosynthesis [87]. Transgenic plants showed a 28% increase 1000-grain weight and an increase in tiller number per plant. Moreover, transgenic plants had a higher fructose, glucose, sucrose and starch contents compared to controls [87]. Furthermore, OE of PFLP resulted in a 2- to 4-fold increase in the transcript levels of CBC enzymes FBPase, SBPase (Figure 1), adenosine diphosphate glucose pyrophosphorylase (AGPase) and sucrose phosphate synthase (SPS) and a corresponding increase in protein levels.

### 2.4. Multi-Targeted Approaches to Improve Photosynthetic Efficiency Can Have a Synergistic Effect

Studies combining the over-expression of CBC proteins, photorespiratory proteins and proteins increasing photosynthetic electron transport have recently been carried out [41,42,88]. These results suggest that manipulating multiple targets can have a synergistic effect on biomass accumulation and that this synergistic effect is target and plant specific (Table 1). These data also demonstrate that targeting different pathways should also be explored. Dual targeting of CBC and photorespiration or CBC and electron transport have resulted in significant increases in yield in both controlled and field experiments (see Table 1). However, targeting of ETC currently focuses on single target manipulation (RieskeFeS, cyt *c*_6_ or ferrodoxin: Figure 2). An approach to increasing electron transport by simultaneously targeting the cyt *b*_6_*f* complex, plastocyanin and ferrodoxin has further potential to either increase electron transport or maintain consistent elevated rates under a range of environmental conditions. Targeting CBC, ETC and Photorespiration concurrently is the next logical step to improve CO_2_ assimilation rates in crops.

Improving photosynthesis to increase yields is currently focused on leaf tissue, however little consideration has been given to the impact of non-foliar green tissues photosynthesis on yields. Recently, Simkin et al. [89] summarized the current literature to evaluate the contribution of different photosynthetically active organs to yield and quality of fruits and grain. Photosynthesis has been shown to occur in in petioles and stems [90], seeds [91], fruit [92,93,94,95] and wheat ears [96] as well as in the husks of corn [97] and it has been suggested that photosynthesis in these tissues could provide an alternative sources of photoassimilates essential for optimal yields and quality. Simkin et al. [89] also showed that transgenic wheat with constitutively increased SBPase activity revealed increased gross photosynthesis in the ears in transgenic lines compared to wild type. These authors further proposed that increases in yield observed in SBPase OE wheat may not be solely due to foliar expression, and that increased activity in the ears may have contributed to the reported 40% increase in grain yield in these plants [89]. This work has highlighted the potential benefits of manipulating non-foliar photosynthesis (i.e., in fruit, wheat ears, seeds and embryos) and their potential impact on quality and nutritional value. 

Therefore, a multi-gene, multi-tissue targeting approach to improving photosynthetic efficiencies in crops may increase the availability of photoassimilates for growth, dependent on (i) the role of non-foliar tissue in photosynthetic assimilation and (ii) the species–species interaction of multiple transgenes on cumulative yield. These areas of exploitation thus require additional research to explore the contribution of non-foliar photosynthesis to yield and quality. Furthermore, as photosynthesis provides the building blocks for a number of vitamin precursors (Figure 1), manipulating photosynthesis offers the potential to modify the nutritional quality of fruit, grain or leaves (see Section 2.5). 

### 2.5. Increasing Photosynthetic Carbon Availability Can Positively Affect Vitamin Content (Biofortification)

More recently, it has been reported that elevated [CO_2_] has a positive effect on Vitamin C (see Section 3.4) accumulation in sour orange (*Citrus aurantium*) [98], strawberry (*Fragaria × ananassa*) [99], tomato [100], and carrots (*Daucus carota*) [101], providing evidence that enhancing CO_2_ assimilation rates through genetic manipulation or growing crops in high [CO_2_] environments (currently used for tomato and strawberry) could potentially impact on Vitamin C content (see Section 3.4). Furthermore, attempts to increase Vitamin C content in plant tissues have had additional outcomes. Vitamin C has been shown to play a role in photo-protection and increasing Vitamin C content in green-tissue has been shown to enhance photosynthetic performance and growth [102]. 

## 3. Biofortification

Biofortification is the process of increasing and concentrating the available micronutrient in crop plants through breeding or genetic engineering. Biofortified crops can be used to improve human nutrition and have the potential to provide the micronutrients required in remote communities with low diet variability. Current approaches to artificially supplement the micronutrient requirements of large populations, either through adding it to food after processing or in pill form, represent a significant and continual financial output. Breeding or genetically engineering can alleviate this need to provide dietary supplementation to large populations over an extended period of time. Nutritionally improved crops can be grown at no additional cost to the farmers and the initial research investment is small compared to the potential economic gains. It has been estimated that every dollar invested in the development of biofortified crops could result is a financial saving of up to $17 [103].

### 3.1. Increasing Pro-Vitamin A Content in Planta 

**Figure 3:**
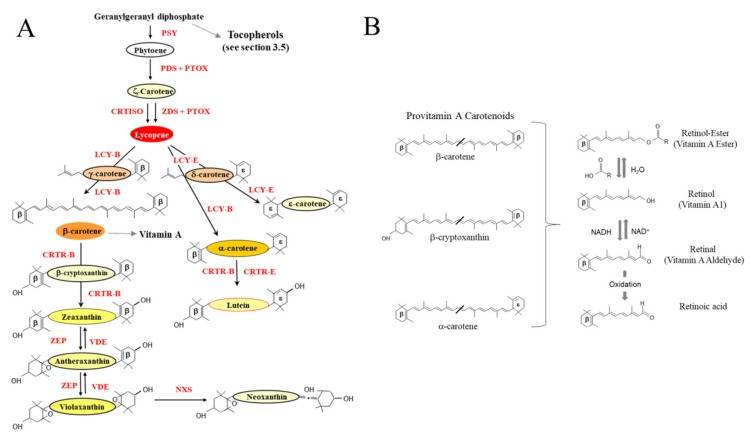
Carotenoid and pro-vitamin A synthesis in planta. (**A**) Overview of the biosynthesis of isoprenoids in plastids. PSY: Phytoene synthase. PDS: phytoene desaturase. ZDS: ζ-carotene desaturase. PTOX: plastid terminal oxidase. CRTISO: carotenoid isomerase. LCY-B: lycopene β-cyclase. LCY-E: lycopene ε-cyclase. CRTR-B: β-carotene hydroxylase. CRTR_E: ε-carotene hydroxylase. ZEP: zeaxanthin epoxidase. VDE: violaxanthin de-epoxidase. NXS: neoxanthin synthase (Adapted from [104,122,123]). (**B**) Carbon structure of the three key provitamin A carotenoids. Beta rings are indicated (β). Retinal is formed either by the enzymatic oxidative cleavage or non-enzymatic degradation of carotenoid precursors (Provitamin A carotenoids). The retinol-ester is primarily consumed from foods of animal origin and is converted to retinol (key storage form) in the small intestine. Retinal can be oxidized to retinoic acid, an essential hormone-like growth factor required by epithelial cells.

Vitamin A is derived from C40 tetraterpenoids (isoprenoids; Figure 1 [104,105]) containing at least one β-ionone ring (see Figure 3A). β-carotene, β-cryptoxanthin and α-carotene are found in green vegetables and fruit. These three carotenoids represent the most common precursors of vitamin A in the human diet and β-carotene is one of the most abundant carotenoids in nature where it plays a key photo-protective role in plants [106,107]. These pro-vitamin A carotenoids are converted into retinal either enzymatically by the β-carotene cleavage oxygenase I, first cloned from fruit flies in 2001 [108] and later from chicken [109] and humans [110] or through non-enzymatic degradation (photodegradation, thermal degradation or oxidation). β-carotene can result in the formation of two molecules of vitamin A and is found in all green leafy plant tissues (i.e., lettuce (*Lactuca sativa*) [111]) and at high levels in some fruits (apricots (*Prunus armeniaca*) [112])) and peach (*P. persica*) [113]) and vegetables (i.e., carrot (*Daucus carota*) [114,115])). 

Vitamin A, also known as retinol, is an essential micronutrient playing important roles in growth and development, vision [116] and the immune system [117]. Without Vitamin A, mammals are incapable of growth, reproduction or of fighting off disease [118]. In the form of retinal (Figure 3B), vitamin A combines with opsin (protein) to form rhodopsin, a light absorbing molecule required for low light and color vision. Retinal can be converted in a reversible reaction to retinol in the small intestine, which functions primarily as a storage form. In the form of retinoic acid, vitamin A functions as an essential hormone-like growth factor for epithelial cells (Figure 3B).

Vitamin A deficiency affects more than a third of all preschool children around the world (and a large proportion of pregnant women) and leads to night blindness and increases the risk of miscarriage and death to pregnant women [119,120]. Most people suffering from a Vitamin A deficiency show no clinical symptoms and people are often unaware of that deficiency in a phenomenon often called ‘hidden hunger’ [121]. Vitamin A deficiencies are more common in populations where staple food crops such as cereals and tubers are relied upon for the vast majority of calories consumed, because these food sources are poor sources of provitamin A carotenoids [121]. Crops such as wheat (*Triticum aestivum*), rice (*Oryza sativa*), cassava (*Manihot esculenta*) and potato (*Solanum tuberosum*), which make up a large part of the diets of poorer communities, contain no significant levels of carotenoids or carotenoid derived products.

Increasing the pro-vitamin A content of these staple crops through genetic engineering of carotenoid biosynthesis has resulted in high carotenoid varieties of tomato (*Solanum lycopersicum*) [124,125,126], maize (*Zea mays*) [127,128], wheat [129], canola (*Brassica napus*) [130], potato (*Solanum tuberosum*) [131,132,133], flaxseed (*Linum usitatissimum*) [134,135], cassava (*Manihot esculenta*) [132] and Sorghum [136,137] (Table 2). Early efforts to increase pro-vitamin A content in rice (*Oryza sativa*) resulted in the generation of the β-carotene enriched “golden rice” [138,139], firstly over-expressing the rate limiting enzyme phytoene synthase (PSY) and subsequently by over-expressing multiple enzymatic steps [139]. Paine et al. [139] also demonstrated that the origin of the PSY was critical to maximising carotenoid accumulation in rice endosperm (Table 2).

Furthermore, enhancing carotenoid deposition by manipulating carotenoid storage sinks has also shown some promise. For example, the over-expression of fibrillin or the Orange Carotenoid protein has been shown to result in a significant increase in carotenoid content in tomato fruit and tubers [126,140,141,142,143,144]. Table 2 further shows the importance of taking a multigene approach to increasing pro-vitamin A content in crops.

It should be noted that increasing carotenoid content to improve pro-vitamin A content has potential drawbacks. Firstly, a 50-fold increase in carotenoids in canola results in (i) an 80% reduction in chlorophyll levels in seeds and (ii) an average 50% decrease in tocopherol levels [130]. The decrease in chlorophyll potentially affects the contribution on non-foliar photosynthesis (see Section 2.5). In canola, seed photosynthesis has been shown to play a role in the accumulation of important storage lipids [150,151] and secondly tocopherol is a required nutrient in human diets (see Section 3.5). The reductions in tocopherol have been addressed in Sorghum, where carotenoid biosynthetic genes were over-expressed along with homogentisic acid geranylgeranyl transferase (HGGT), which catalyses the committed step of tocotrienol biosynthesis has previously been shown to enhance total vitamin E antioxidants (see Section 3.5) (tocotrienols plus tocopherols) content by 10- to 15-fold [152]. Furthermore, in cassava and potato, increasing content resulted in a ~25% decrease in the dry matter content of the tubers, and a decrease in starch content, whist sucrose, glucose, total fatty acid, triacylglycerols increased [132].

Biofortified maize engineered to accumulate pro-vitamin A has shown to be effective at significantly increasing the total body stores of vitamin A in 5- to 7-year-old children [153]. Consumption of β-carotene fortified maize has been proven to be as effective at controlling vitamin A deficiency as taking supplements [153] and has also been shown to improve visual function in children with a vitamin A deficiency [154]. Although these results clearly show the benefits of increasing pro-vitamin A content in staples, these increases come with metabolic and nutrient changes that merit more investigation. 

### 3.2. Genetic Manipulation of Folate (Vitamin B_9_) Accumulation

**Figure 4:**
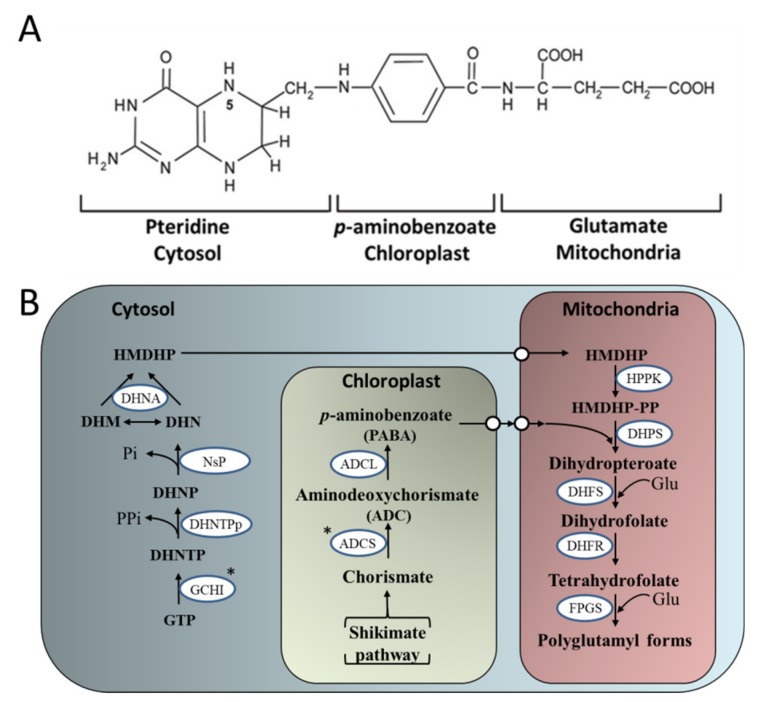
The plant folate biosynthesis pathway. (**A**) The chemical structure of monoglutamyl tetrahydrofolate (**B**) The pteridine pathway leading to hydroxymethyldihydropterin (HMDHP) in the cytosol; the pathway leading to p-aminobenzoate in the plastid, and condensation steps localized in the mitochondria are shown. DHN, dihydroneopterin; -P, monophosphate; -PP, pyrophosphate; -PPP, triphosphate; DHM, dihydromonapterin; Glu, glutamate; ADCS, aminodeoxychorismate synthase; ADCL, aminodeoxychorismate lyase; GCHI, GTP cyclohydrolase I; DHNTPp, dihydroneopterin triphosphate pyrophosphatase; NsP, non-specific phosphatase; DHNA, dihydroneopterin aldolase; HPPK, hydroxymethyldihydropterin pyrophosphokinase; DHPS, dihydropteroate synthase; DHFS, dihydrofolate synthase; DHFR, dihydrofolate reductase; FPGS, folylpolyglutamate synthetase. White circles represent points of substrate transport. *targeted enzymes for over-expression (Adapted from [155,156]).

Folate is synthesized by plants and micro-organisms, is water soluble and is an essential micronutrient. Folates consist of three parts, a pteridine, p Aminobenzoate (PABA), and glutamate moieties (Figure 4A). In plants, folate biosynthesis is compartmentalized, with PABA synthesized in the plastid (derived from chorismate [from Shikimate pathway, Figure 1]) and pteridines synthesized from guanosine-50-triphosphate (GTP) in the cytosol. These two moieties are translocated into the mitochondria, where they are condensed to form dihydropteroate, which is then glutamylated (modified by reaction with glutamate (Glu)) to form folates (Figure 4B) [155,156]. In plants, folates play essential roles in photorespiration, and in the synthesis of chlorophyll, plastoquinone, tocopherol, making them essential for plant health and development [157]. 

It has been recommended that adults consume 400 mg of folate from foods or dietary supplements daily with pregnant women needing as much as 600 mg daily [158]. Folates are essential coenzymes and a folate deficiency during pregnancy can cause neural tube defects (NTDs) in infants (i.e., spina bifida and anencephaly) and megaloblastic anemia [159,160,161]. In adults, folate deficiency has been associated with an increased risk of cardiovascular and coronary diseases, some forms of cancers and a loss of cognitive function [162,163]. Unfortunately, many crops consumed specifically by at risk populations (poorer communities in developing countries), including wheat, potatoes, cassava, rice and maize, contain insufficient quantities of folate and are unable to meet daily requirements, especially where other types of food are limited. Currently, flour and cereal-grain products are fortified with folic acid to overcome the issues of folate deficiency within the population; however, this does not address the issues of folate deficiency in poorer communities. To overcome this, genetic engineering approaches have been applied to increase folate content in crops [164,165,166]. 

Over-expression (OE) of the GTP cyclohydrolase I (GCHI) (Figure 4B), the first committed step in the pteridine pathway [167], in Arabidopsis resulted in a >1000-fold increase in pterins and only a 2- to 4-fold increase in folates (Table 3) [166]. Similar results were also observed following Seed-specific over-expression of GCHI in common Mexican bean [168]. These results were supported by similar work carried out by Diaz de la Gaza [164] who demonstrated that the OE of GCHI in tomato fruit resulted in an up to 140-fold increase in pterins and increased fruit folate content by an average of 2-fold [164]. In tomato fruit, OE of GCHI was also shown to result in the depletion of the chloroplast synthesized PABA, which suggested that PABA represents a further limiting step in folate biosynthesis in transgenic tomato fruit (Table 3). However, this depletion in PABA is in contrast with an increase observed following the OE of GCHI in Mexican bean [168]. These authors demonstrated that feeding fruit PABA though the fruit stalk could increase folate by up to 10-fold [164]. 

A further attempt to increase fruit folate content came from the OE of the plastid localized aminodeoxychorismate synthase (ADCS) (Figure 4B), resulting in an up to 19-fold increase in PABA levels in ripe fruit. However, the OE of ADCS did not lead to an accumulation of folate and no differences could be observed between OE lines and controls (Table 3) [165]. However, these same authors demonstrated that when ADCS-OE lines were crossed with GCHI-OE lines, these double ADCS/GCHI transgenic lines accumulated pterins, PABA and folate in red ripe fruit. Work in other species has also demonstrated that increasing folate concentration by metabolic engineering remains an important direction to produce healthier fortified crops including staple crops consumed in developing countries (Table 3).

Work carried out by Dong et al. [169] in rice has shown that the manipulation of other folate biosynthetic genes, individually or in combination, can also lead to small increases in folate. This potential opens the door to a multigene approach to increasing folate concentration in a variety of foods. Recently, the bioavailability of metabolically engineered folate-biofortified rice was demonstrated in rats [170]. Studies evaluating this cost-effective approach to folate supplementation in China [171] have suggested that folate-biofortified crops could be invaluable in addressing the incidence of folate deficiency in whole populations [172].

### 3.3. Cobalamin (Vitamin B_12_) 

Cobalamin, the biologically active form of Vitamin B_12_ (B_12_), is part of the modified tetrapyrrole family that includes molecules such as chlorophyll and haem and is made exclusively by a small group of prokaryotes (bacteria and archaea) [176]. Some of these bacteria are found in the flora of ruminant mammals where they proliferate in the stomach and continue to form B_12_. This source of B_12_ accumulates in animal product including meat, eggs, milk [177] and is the key dietary source of B_12_ in the population, which raises issues for those following a vegan diet requiring supplementation often in the form of fortified cereals, plant-based milks, nutritional yeast, tablets or intramuscular injection [178] in order to avoid serious health consequences. B_12_ is absent from fruits and vegetables, although nori, an edible purple/green, contains a significant concentration of B_12_ [177]. However, this source of B_12_ is controversial due to the presence of high quantities of pseudovitamin B_12_, which may not be bioavailable and is also an inactive analogue in human diets [177].

The recommended daily allowance of B_12_ for adult men and women is 2.0–2.4 µg/day with 2.6 µg/day for pregnant women. Vitamin B_12_ is an essential nutrient for animals where it is a cofactor required for the synthesis of DNA and neurotransmitters as well as for fatty acid and amino acid metabolism. Even at levels slightly lower than normal, B_12_ deficiency can result in a range of symptoms including depression, loss of memory (reduced cognitive performance), fatigue, lethargy and headaches and in some people mania and psychosis [179,180]. Many of these symptoms are the result of pernicious anaemia, a lack of red blood cells, which can lead to additional symptoms including chest pain, numbness in the hands and feet, poor reflexes, smooth red tongue and a shortness of breath [181].

**Figure 5:**
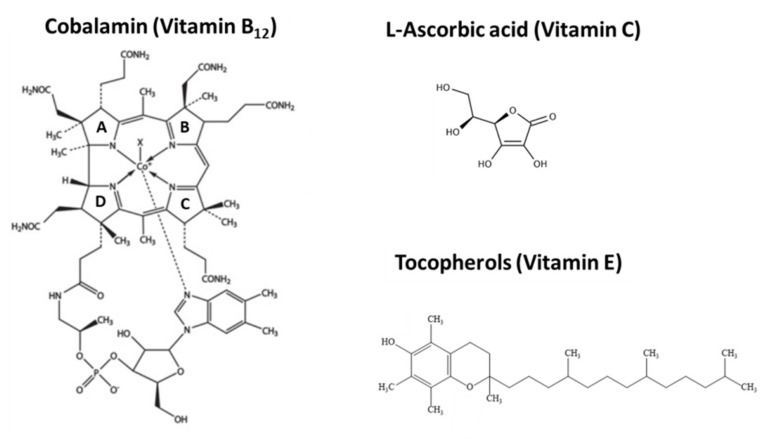
The chemical structures of Vitamin B_12_ (pyrrole rings are labelled A to D); Vitamin C and Vitamin E.

Cobalamin is the most structurally complex and largest vitamin containing a cobalt at its centre surrounded by four pyrrole rings (Figure 5) and its biosynthesis requires approx. 30 enzyme-mediated steps, including a number of oxygen-sensitive and highly labile intermediates that make its synthesis by eukaryotic cells highly challenging [182,183,184]. 

Due to the highly complex nature of B_12_, work to biofortify crops through genetic engineering has not yielded any significant results. However, biofortification of plants via feeding mechanisms has demonstrated that *Lepidium sativum* (garden cress) can take up B_12_ if grown in B_12_ enriched media, where it accumulates in the vacuole of the cotyledons, although the mechanism of uptake remains to be elucidated [185]. Furthermore, this method of biofortification requires the use of B_12_ from other sources, such as bacterial fermentation cultures [186], and due to the ever-increasing price of vitamin B_12_ supplements this may not prove to be a viable option for biofortifying crops on a large scale unless a significant increase in vitamin B_12_ manufacturing facilities is undertaken. 

### 3.4. L-ascorbic Acid (Vitamin C)

Humans and some other primates have lost the ability to synthesize and store Vitamin C and depend on dietary provision to cover their daily requirements of 75–90 mg. Vitamin C (Figure 5) is found in citrus fruits (oranges and lemons, etc.), grapefruit, mango, kiwifruit, broccoli, Brussels sprouts, tomatoes and a variety of berry fruits (strawberries, raspberries, blueberries, and cranberries) [187]. Vitamin C is considered to be an essential nutrient required for the repair of connective tissues, collagen synthesis and the enzymatic production of some neurotransmitters. It is also important for correct immune system function and acts as an antioxidant. Previous research has suggested that Vitamin C can aid in the treatment of Alzheimer’s, Huntington’s and Parkinson’s disease [188,189,190].

Under physiological pH conditions, Vitamin C exists almost exclusively as an ascorbate anion and functions as a co-factor for methylcytosine dioxygenases, enzymes involved in the demethylation of DNA [191]. Furthermore, Ascorbate serves as a cofactor for the Jumonji C-domain-containing histone demethylases required for the demethylation of histone [191]. These authors suggested that by participating in the demethylation of both DNA and histones, Vitamin C regulates epigenetic processes mediating the interaction between the environment and the genome. The impact of this interaction on human health has been reviewed [192]. In short, ascorbate deficiency potentially affects neonatal and postnatal developmental processes with long-term consequences for health through epigenetic dysregulation (i.e., cancer and neurodegeneration). Therefore, Vitamin C deficiency, in addition to the immediate consequences to the mother, could have long-term consequences for unborn children.

Vitamin C is well tolerated and often taken as a supplement in tablet form, although in large doses Vitamin C can cause gastrointestinal discomfort, headache and result in insomnia. However, at high concentration it has been reported to play a role in the treatment of cancer, arteriosclerosis, and a number of other cardiovascular diseases [193,194,195]. In plants, Ascorbate metabolism is strongly linked with photosynthesis and has a well-documented role in photoprotection [196,197]. Ascorbate is a major antioxidant in chloroplast and has been reported to be an important cofactor for VDE activity in the carotenoid biosynthetic pathway (Figure 3A) [196]. When the absorption of light is beyond the plant’s capacity, the excess energy needs to be dissipated. VDE converts violaxanthin (V) to zeaxanthin (Z) in the daytime and zeaxanthin epoxidase (ZEP) converts Z back to V at night [198]. This inter-conversation, known as the xanthophyll cycle, is responsible for the dissipation of excess energy protecting photosynthetic membranes. 

**Figure 6:**
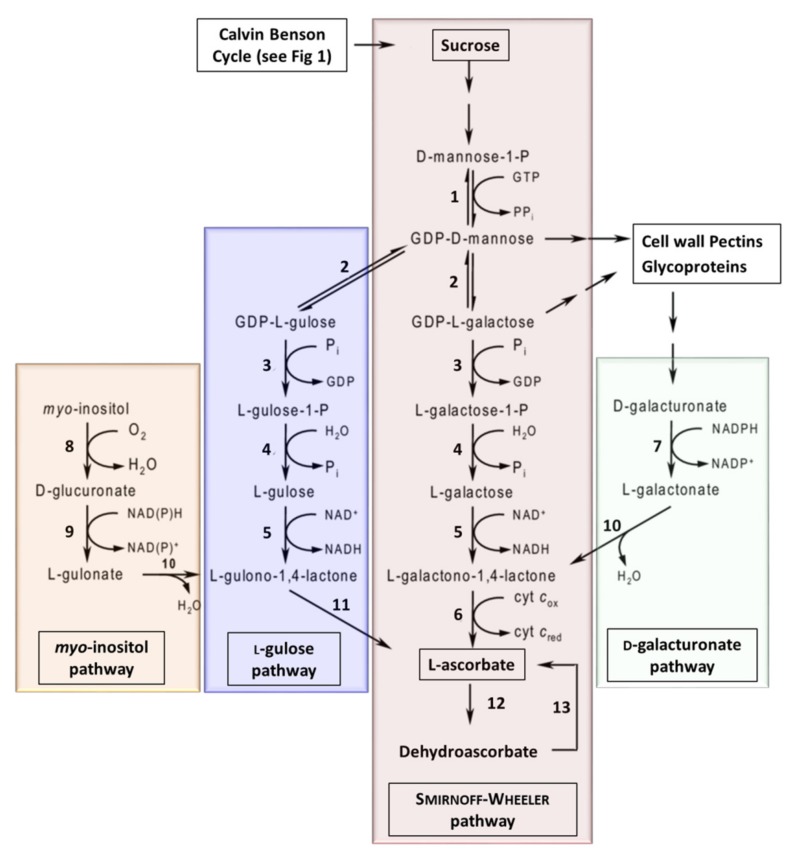
The biosynthetic pathways for vitamin C in plants. Enzymes: 1, GDP-D-Man pyrophosphorylase; 2, GDP-Man-3’,5’-epimerase; 3, GDP-L-Gal phosphorylase (or transferase); 4, L-Gal 1-phosphate phosphatase; 5, L-Gal dehydrogenase; 6, L-GalL dehydrogenase; 7, D-galacturonate reductase; 8, myo-inositol oxygenase; 9, D-glucuronate reductase; 10, aldonolactonase; 11, L-GulL oxidase (or dehydrogenase); 12, ascorbate oxidase; 13, dehydroascorbate reductase (adapted from [149,208]).

In plants, several different ascorbate biosynthesis pathways have been characterized (Figure 6). The Smirnoff–Wheeler pathway is the primary pathway in plants [199], however, three ‘alternative’ pathways have also been shown to lead to the production of ascorbate in planta. The myo-inositol pathway [200], L-gulose pathway [201,202], and D-galacturonate pathway [203,204]. However, work using Arabidopsis mutants suggests that these alternative pathways contribute a relatively small amount of ascorbate to the overall pool [205,206]. It has been suggested that manipulating these pathways to increase Vitamin C, to improve nutritional quality of horticultural crops, is a clear target of future research [207]. A number of reports have evaluated the over-expression or down-regulation of genes to determine their impacts on ascorbate levels (Table 4). The reports have focused either on the over-expression of biosynthesis and recycling enzymes or antisense suppression of ascorbate oxidase (enzyme 12: Figure 6 and Table 4).

Over-expression of genes GDP-Man-3’,5’-epimerase ((GME) enzyme 2: Figure 6 and Table 4), a key step in both the Smirnoff–Wheeler and L-gulose pathways, was shown to result in a 1.2- to 1.6-fold increase in ascorbate in tomato fruit [209]. Furthermore, over-expression GDP-L-Gal phosphorylase ((GMP) enzyme 3: Figure 6 and Table 4), which is also active in both the Smirnoff–Wheeler and L-gulose pathways, was shown to result in a 2- to 6-fold increase in ascorbate levels in strawberry and tomato fruit, respectively, and an up to 3-fold increase in ascorbate in potato tubers [210]. GDP-L-Gal phosphorylase ((GGP) enzyme 3: Figure 6) over-expression increased ascorbate content, suggesting that GGP is a key enzyme in the Smirnoff–Wheeler pathway [211]. Other studies have demonstrated that over-expression of GDP-D-Man pyrophosphorylase ((GMP) enzyme 1: Figure 6) also facilitates an increase in ascorbate [212]. 

In 2019, the over-expression of L-Gal dehydrogenase ((GDH) enzyme 5: Figure 6) or L-GalL dehydrogenase ((GLDH) enzyme 6: Figure 6) also led to small increases in ascorbate content in acerola fruits (*Malpighia glabra*), whilst the over-expression of GME had minimal effect on ascorbate levels [219]. These authors also demonstrated that the co-expression of GMP (enzyme 1) and GGP (enzyme 3) resulted in higher ascorbate levels compared to GMP or GGP alone [219]. Furthermore, co-overexpression of GMP (enzyme 1), GME (enzyme 2), and GGP (enzyme 3) significantly increased ascorbate contents compared to the dual expression of GMP and GGP [219]. These data suggest that expressing the three upstream enzymes in the Smirnoff–Wheeler pathway has a synergistic effect on ascorbate levels. In contrast, co-overexpression of GGP (enzyme 4), GDH (enzyme 5), and GLDH (enzyme 6) resulted in no differences in ascorbate [219]. 

A number of reports have also evaluated the over-expression of targets in the three ‘alternative’ ascorbate biosynthetic pathways. For example, the over-expression of L-gulonolactone oxidase (enzyme 11: Figure 6) results in a 2- to 7-fold increase in ascorbate levels [217,218]. Furthermore, over-expression of D-galacturonate reductase (enzyme 7: Figure 6) resulted in a 2- to 3-fold increase in ascorbate in Arabidopsis [203]. Finally, constitutive expression of myo-inositol oxygenase (enzyme 8: Figure 6), which catalyzes the oxidation of myo-inositol into D-glucuronate, resulted in a 2- to 3-fold increase in ascorbate (Table 4) [200]. 

Some horticultural crops, including tomato and strawberry, are commercially grown at elevated CO_2_ under glass. Elevated [CO_2_] has previously been shown to cause an increase in ascorbate in sun-acclimated leaves of sour orange [220], and recent work by Wu et al. has shown that carrots grown under elevated [CO_2_] accumulate Vitamin C due to changes in the expression levels of 12 biosynthetic genes [101]. Furthermore, elevated [CO_2_] was also shown to result in increases in the expression of ascorbate-related genes and enzyme activities and a 1.09–3.91-fold increase in Vitamin C in celery petioles [221]. These results suggest that enhancing photosynthesis (see Section 2) is a potential route to biofortify crops. However, it should be noted that growing barley at elevated [CO_2_] resulted in a significant decrease in ascorbate [222], demonstrating species–species response differences to atmospheric CO_2,_ which requires further study. For review, see [187]. Finally, down-regulating the activity of the mitochondrial malate dehydrogenase in transgenic tomato was shown to enhance photosynthesis, chloroplastic electron transport rates and CO_2_ assimilation, resulting in an 11% and 19% increase in dry matter and a 5.7-fold increase in ascorbate in fruit (Table 4) [102]. In addition to the results obtained from growth in high CO_2_, this supports the idea that enhancing CO_2_ uptake can positively influence the Vitamin C content of foods. 

### 3.5. Tocopherols (Vitamin E)

Vitamin E is a fat soluble group of compounds including four tocopherols and four tocotrienols, the most active of which is α-tocopherol (Figure 5). It functions to protect cell membranes from reactive oxygen species, the result of which can be nerve damage. Furthermore, vitamin E deficiency is responsible for a number of conditions including impairment of immune responses, myopathies, retinopathy, peripheral neuropathy and ataxia (progressive neurodegenerative disorder). However, Vitamin E deficiency is humans is rare and is usually the consequence of a metabolic disorder rather than a lack of Vitamin E in the diet. To enhance Vitamin E content in Corn, Cahoon et al. [152,223] over-expressed cDNAs encoding homogentisic acid geranylgeranyl transferase (HGGT), resulting in a 10- to 15-fold increase in total vitamin E antioxidants (tocotrienols plus tocopherols). This was further demonstrated in Sorghum by Che et al. [136] who over-expressed HGGT in combination with the over-expression of cDNA encoding carotenoid biosynthetic enzymes (Table 2). These authors demonstrated that with this combination of genes, transgenic sorghum could accumulate all-trans β-carotene (~19-fold increase see Section 3.1) and a 1.8-fold and 1.7-fold increase in α-tocopherol and γ-tocopherol, respectively, and a 27-fold increase in α-tocotrienol [136].

### 3.6. A Multi-Targeted Approach to Multi-Vitamin Crops

Previous studies have used multigene targeted approaches to increase the nutritional content of various crops; however, Naqvi et al. [149] used a multigene, multivitamin approach to boost three vitamins from three distinct metabolic pathways, namely Vitamin C, folate and provitamin A in South African Corn. Naqvi et al. over-expressed the *Pantoea ananatis* carotene desaturase (CRTI) and Maize psy (Figure 3A) to increase β-carotene (provitamin A) from 0.35 µg/g DW to 15–59 µg/g DW in the best lines and up to a 100-fold increase in total carotenoids including increases in α-carotene and β-cryptoxanthin, which also serve as substrate for vitamin A (Table 2; see Section 3.1). In the same plants, these authors expressed the *Esherichia. coli* folE, encoding GTP cyclohydrolase I (GCHI: Figure 4B) and the rice dehydroascorbate reductase (DHAR: Enzyme 13: Figure 6) to increase folate and vitamin C content by ~2-fold (Table 3; see Section 3.2) and up to 7.5-fold, respectively (Table 4; See Section 3.4).

## 4. Future Prospects and Conclusions

With increasing requirements for food and fuel, the need to develop varieties of important crops with greater yields is at the forefront of agriculture. In recent years, using genetic manipulation to increase the rate of photosynthesis as a means of increasing yields has also gained traction. Evidence supporting this has come from both modelling approaches and from initial work looking at the over-expression of single genes in a variety of transgenic plants. Later, a multi-targeted approach to genetic manipulation further supported the idea that manipulating multiple targets in CBC, photorespiration and electron transport could have a synergistic effect resulting in greater increases in yield. Furthermore, growing carrots, strawberry, tomato, celery and citrus in elevated [CO_2_] resulted in an increase in vitamin C content, demonstrating that increasing CO_2_ assimilation has the potential to increase nutritional content. These data also suggest that manipulating photosynthesis to improve CO_2_ assimilation without increasing atmospheric [CO_2_] could potentially also result in increases in plant vitamin content. However, this situation may be more complex and species specific. Cober et al. [224] reported that growing soybean in elevated [CO_2_] resulted in an increase in seed yield but seed oil concentrations and seed protein levels were reduced. Furthermore, a meta-analysis carried out by Myers et al. [225] reported that C_3_ plants and legumes grown under elevated [CO_2_] had lower concentrations of zinc and iron; and in non-legume C_3_ plants a reduction of protein content. Zhu et al. [226] confirmed these results and also demonstrated declines in vitamins B1, B2, B5, and B9 in Rice grown under high [CO_2_]. Interestingly, these authors also observed an increase in vitamin E (see Section 3.5) in these same plants. With global increases in atmospheric [CO_2_], temperature and rainfall, these results demonstrate that climate change could significantly impact the nutritional quality of our crops. 

Biotechnology programs are at the forefront of agricultural research and adopting techniques such as genetic engineering and genome editing for endogenous genes manipulation [227,228,229] is key to tackle these issues. To generate plants with sustainable increases in yields and nutritional quality, and with the ability to adapt successfully to changing environmental conditions, will require a multi-targeted approach touching upon multiple aspects of carbon assimilation, light adaptation and nutrient biosynthesis and will require new tools including vectors for multiple gene insertion [230,231,232,233,234] and tissue-specific promoters [235,236,237,238,239,240]. If the promise of these biotechnology programs is to be realized, public perception of genetic modification and genome editing technologies will need to be addressed. ‘Golden Rice’, for example, biofortified with pro-vitamin A, was engineered by a team of European scientists with the hope of combatting premature blindness and early death caused by vitamin A deficiencies in human populations that subsist on nutrient poor white rice. However, 20 year later, Golden rice has failed to reach these populations and it has been reported that tens of millions of people in countries including China, Bangladesh, and South and Southeast Asia have died or gone blind [241]. Golden rice has been described by critics as a ‘hoax’, as ‘fool’s gold’ and as propaganda for GM technologies and supporters have described the 20 years delay in its introduction as a crime against humanity [241]. Finally, all countries have in place, regulatory mechanisms for the approval of GM crops; however, these differ significantly and are often complicated by non-science based systems [242,243,244]. A more streamlined long-term approach may be needed for international adoption of these technologies. 

Previous work has shown that manipulating the expression levels of genes for provitamin A, Folate and vitamin C metabolism can result in an increase in these micronutrients and that combining these manipulations results in multivitamin corn. This research opens the avenue of manipulating photosynthesis and vitamin metabolism to create high yielding multivitamin crops for addressing ‘hunger’ and ‘hidden hunger’ in at risk populations.

## Figures and Tables

**Table 1:** Summary of the cumulative impacts of multiple transgenes manipulating Calvin–Benson Cycle, Photorespiration, CO_2_ transport and Photosynthetic Electron Transport and their biological outcomes (Arabidopsis; LL; Low Light = 130 mol/m^−2^/s^−1^ and HL; High Light = 390 mol/m^−2^/s^−1^. Tobacco LL = 200–350 mol/m^−2^/s^−1^; HL = 600–1400 mol/m^−2^/s^−1^). Sedoheptulose-1,7-bisphosphatase (SBPase); fructose-1,6-bisphosphate aldolase (Ald); Bifunctional FB/SBPase (FS Bif); Glycine decarboxylase H protein (GDCH) Cytochrome *c*_6_ (Cyt *c*_6_); inorganic carbon transporter B (ictB) (see Figure 1 and Figure 2).

Manipulation	Plant	Transgene(s) Expressed			Biomass and Yield	Ref
CBC and Photorespiration	Arabidopsis Col-0	SBPase	-	-	42% increase in dry weight. 53% increase in seed yield under LL (39% increase in seed yield under HL)	[41]
		-	Ald	-	32% increase in dry weight. 35% increase in seed yield under LL (36% increase in seed yield under HL)	
		SBPase	Ald	-	41% increase in dry weight. 49% increase in seed yield under LL (20% increase in seed yield under HL)	
		-	-	GDCH	50% increase in dry weight. 0% increase in seed yield under LL (0% increase in seed yield under HL)	
		SBPase	Ald	GDCH	71% increase in dry weight. 42% increase in seed yield under LL (62% increase in seed yield under HL)	
CBC and CO_2_ transport	Tobacco cv Samsun	SBPase	-	-	30–34% increase in dry weight under HL (52% under LL)	[42]
		-	-	ictB	71% increase in dry weight (HL)	
		SBPase	-	ictB	92% increase in dry weight under HL (76% under LL)	
		SBPase	Ald	-	62% increase in dry weight under HL (54% under LL)	
		SBPase	Ald	ictB	103% increase in dry weight under HL (79% under LL)	
CBC and CO_2_ transport	Rice	FS Bif	-	-	no increase in biomass observed	[88]
		-	-	ictB	no increase in biomass observed	
		FS Bif	-	ictB	increase in biomass demonstrating the synergistic effect	

**Table 2:** Summary of the cumulative impacts of multiple transgenes manipulating the accumulation of pro-vitamin A carotenoids (See Figure 3A). 1-Deoxy-D-xylulose-5-phosphate synthase (*Dxs*); phytoene synthase (Psy) phytoene desaturase (Pds); lycopene β-cyclase (Lyc); Orange carotenoid protein (Or); Fibrillin (Fib); *Hordeum vulgare* homogentisic acid geranylgeranyl transferase (HGGT); *Erwinia uredovora* phytoene synthase (crtB); *Erwinia uredovora* phytoene desaturase (crtL); *Pantoea ananatis* carotene desaturase (CrtI); *E. uredovora* lycopene β-cyclase (CrtY); *Escherichia coli* phosphomannose isomerase (PMI); *E. coli* 1-Deoxy-D-xylulose-5-phosphate synthase (DXS).

Plant	Transgene(s) Expressed			Metabolite Analysis	Ref
Tomato fruit	crtB	-	-	phytoene content increase (1.6–3.1-fold). Lycopene (1.8–2.1-fold) and β-carotene (1.6–2.7-fold) were increased	[125]
	-	crtL	-	β -carotene content increased about threefold, up to 45% of the total carotenoid content	[124]
	-	-	SlLyc	Increase in total carotenoids (2.3-fold). β-carotene increased (11.8-fold) and Lycopene decrease (10-fold)	[145]
	-	-	AtOr	Increases in Lycopene (1.6-fold), α-carotene 2.6-fold) and β-carotene (2.7-fold)	[142]
	-	-	CaFib	Increases in Lycopene (2.2-fold) and β-carotene (1.6-fold)	[126]
Cassava tubers	crtB	-	-	~15-fold increases in carotenoids (as all-trans-β-carotene) (40–60 µg/g DW compared to CN 0.5–1 µg/g DW)	[132]
	crtB	AtDxs	-	20- to 30-fold increases in carotenoids (as all-trans-β-carotene) (25 µg/g DW) compared to CN 0.5–1 µg/g DW)	
	-	-	BoOr	~2-fold increases in carotenoids (as all-trans-β-carotene) (3–4 µg/g DW) compared to CN 0.5–1 µg/g DW)	
Potato tubers	-	DXS	-	2-fold increase in total carotenoids and 6- to 7- fold increase in phytoene	[146]
	crtB	-	-	Carotenoid levels reached 35 μg/g. β-carotene levels in the transgenic tubers reached ~11 μg/g DW	[133]
	crtB	AtDxs	-	37–109 µg/g DW total carotenoids (CN 8 µg/g)	[132]
	crtB	crtL	crtY	20-fold increase (to 114 µg/g DW) with β-carotene 3600-fold higher (47 µg /g DW)	[131]
	-	-	BoOr	The total carotenoid contents were 6-old higher than CN. Increasing from ~4 µg/g DW to ~22 µg/g DW	[140]
Canola seed	crtB	-	-	50-fold increase in carotenoids with α- and β-carotene. Lutein, the predominant carotenoid in CN seeds remained at similar levels in transgenic seeds	[130]
Soybean	crtB	-	-	Accumulate 845µg/g DW of β carotene. An increase of 1500-fold compared to CN	[147]
Wheat	ZmPsy	ctrI	-	Increase β-carotene from 0.81µg /g DW to 2.3–4.9 µg /g DW in the best lines	[129]
Cavendish Banana	MtPsy	-	-	Increase in β-carotene content from 3.1 µg/g DW in fully ripe fruit to up to 8.3 µg/g DW.	[148]
	ZmPsy	-	-	Increase in β-carotene content from 3.1 µg/g DW in fully ripe fruit to up to 9.0 µg/g DW.	
	ZmPsy	ctrI	-	Increase in β-carotene content from 3.1 µg/g DW in fully ripe fruit to up to 13.2 µg/g DW.	
Maize	ZmPsy	ctrI	-	Increase β-carotene from 0.35 µg /g DW to 15–59 µg /g DW in the best lines. Up to 100-fold increase in total carotenoids (see Section 3.6)	[149]
	crtB	ctrI	-	Increase β-carotene from 0.39 µg /g DW to 9.8 µg /g DW in the best line	[127]
Rice	NpPsy	crtI	-	β-carotene, + small amounts of lutein and zeaxanthin	[138]
	NpPsy	crtI	NpLyc	1.6 µg/g carotenoid in the endosperm	
	NpPsy	crtI	-	0.8–1.2 µg/g (up to 68% β-carotene)	[139]
	SlPsy	crtI	-	0.9–1.2 µg/g (up to 68% β-carotene)	
	CaPsy	crtI	-	1.1–4.7 µg/g (up to 80% β-carotene)	
	ZmPsy	crtI	-	Up to 14.5 µg/g (up to 89% β-carotene)	
	OsPsy	crtI	-	Up to 18.4 µg/g (up to 86% β-carotene)	
Sorghum	AtDxs	ZmPsy	ctrI, PMI	β-carotene levels ranged from 2.5 to 9.1 μg/g DW in the mature seeds compared to CN 0.5 μg/g DW (+10-fold)	[136]
	HGGTAtDxs	ZmPsy,	ctrI, PMI	all-trans β-carotene levels ranged from 7.3 to 12.3 μg/g DW in the mature seeds compared to CN 0.5 μg/g DW (~19-fold increase) +1.8-fold increase in α-tocopherol	

Oryza sativ (Os); Solanum lycopersicum (Sl); Capsicum annum (Ca); Brassica oleracea (Bo); Arabidopsis thaliana (At); Zea mays (Zm); Narcissus pseudonarcissus (Np); Hordeum vulgare (Hv); Musa troglodytarum x acuminate (Mt). CN = control.

**Table 3:** Summary of the cumulative impacts of multiple transgenes manipulating folate metabolism. NR = not reported. CN = control. GTP cyclohydrolase I (GCHI); aminodeoxychorismate synthase (ADCS) (see Figure 4B).

Plant	Transgene(s) Expressed		Metabolite Analysis			Ref
			Pterins	PABA	Folate	
Arabidopsis	GCHI	-	1250-fold increase	NR	2- to 4-fold increase.	[166]
Mexican Bean	GCHI	-	150-fold increase	Increase	Up to 3-fold increase in desiccated beans	[168]
Lettuce	GCHI	-	NR	NR	2- to 8.5-fold increase	[173]
Potato	GCHI		Approx. 18-fold increase	Decrease	Up to 2-fold increase	[174]
	GCHI	ADCS	Approx. 33-fold increase	>6-fold increase	Up to 3-fold increase	
Tomato Fruit	GCHI	-	3- to 140-fold increase	Severely depleted	average 2-fold increase in ripe fruit	[164,165]
	-	ADCS	No increase observed	Up to 20-fold increase	No increase observed in ripe fruit	
	GCHI	ADCS	Up to 30-fold increase	Up to 20-fold increase	Up to 25-fold increase in ripe fruit	
Rice	GCHI	-	25-fold increase	NR	No increase observed	[175]
	-	ADCS	NR	49 times higher than controls	6 times lower than in controls	
	GCHI	ADCS	4-fold increase	25 times high than control	15–100 times higher than CN	
Corn	*E. coli* folE encoding GCHI		NR	NR	~2-fold increase (see Section 3.6)	[149]

**Table 4:** Summary of the impacts of manipulating ascorbate biosynthetic enzymes on ascorbate accumulation and ascorbate dehydroascorbate ratios (see Figure 6).

Plant	Enzyme	Regulation	Metabolite Analysis	Ref
Tomato	GDP-Man-3’,5’-epimerase	Up	1.2- to 1.6-fold increase in fruit	[209]
Arabidopsis	GDP-L-Gal phosphorylase	Up	Up to 4-fold increase in best lines	[211]
Tomato	GDP-L-Gal phosphorylase	Up	3- to 6-fold increase in fruit ascorbate	[210]
Strawberry	GDP-L-Gal phosphorylase	Up	2-fold increase in tuber ascorbate	[210]
Potato	GDP-L-Gal phosphorylase	Up	Up to 3-fold increase in fruit ascorbate	[210]
Tobacco	ascorbate oxidase	Down	1.9-fold increase in ascorbate and increase in the Ascorbate to DHA ratio	[213]
Tobacco	ascorbate oxidase	Down	No increase in the ascorbate pool but increase in ratio of Ascorbate to DHA	[214]
Tobacco	dehydroascorbate reductase	Up	2.2- to 3.9-fold increase in ascorbate and increase in the Ascorbate to DHA ratio	[215]
Maize	dehydroascorbate reductase	Up	1.9-fold increase in ascorbate and increase in the Ascorbate to DHA ratio	[215]
Arabidopsis	D-glucuronate reductase	Up	2- to 3-fold increase in ascorbate	[203]
Potato	D-galacturonate reductase	Up	Up to 2-fold increase in tuber ascorbate	[216]
Tobacco	L-gulonolactone oxidase	Up	7-fold increase in ascorbate	[217]
Lettuce	L-gulonolactone oxidase	Up	4- to 7-fold increase in ascorbate	[217]
Arabidopsis	L-gulonolactone oxidase	Up	~2.0-fold increase in ascorbate	[218]
Arabidopsis	myo-inositol oxygenase	Up	2- to 3-fold increase in the ascorbate content of leaves compared with controls	[200]
Corn	dehydroascorbate reductase	Up	Up to 7.5-fold increase in ascorbate (see Section 3.6)	[149]
Tobacco	malate dehydrogenase	Down	5.7-fold increase in ascorbate	[102]
